# Electrocardiographic ST-segment elevation with prominent R waves in precordial leads—an alternative explanation

**DOI:** 10.1007/s12471-022-01748-x

**Published:** 2022-12-19

**Authors:** A. P. M. Gorgels

**Affiliations:** 1 Department of Cardiology, Maastricht University Medical Centre+, Maastricht, The Netherlands; 2 HartKliniek Maastricht, Maastricht, The Netherlands

Dear editor,

With interest, I read the Rhythm Puzzle and the accompanying question and answer titled ‘Electrocardiographic ST-segment elevation with prominent R waves in precordial leads’ by Andreou and Pérez-Riera in a recent issue of the *Netherlands Heart Journal* [1, 2]. The authors present a case of acute anterior wall myocardial infarction with tall R waves in the precordial leads. They argue the following: (1) the increase in R wave voltage, compared with the nonischaemic preceding electrocardiogram (ECG), was caused by left septal fascicular block (LSFB); (2) of all causes of prominent anterior QRS forces, only LSFB can manifest as intermittent changes; and (3) although the culprit stenosis is clearly located after the first septal branch of the left anterior descending coronary artery, spasm of that branch led to the observed ECG changes.

I kindly disagree with these interpretations. Herein, I offer an alternative explanation and discuss some aspects of the patient’s ECG that are not or incompletely discussed by the authors.

The ECG during ischaemia shows ST-segment elevation in leads II, III and aVF, meaning that the frontal ST vector points inferiorly, which is indicative of an occlusion distal from the first septal and first diagonal branch [3]; this is exactly what can be seen on the coronary angiogram (see Fig. 2 in the ‘Answer’ section) [2]. It precludes involvement of the first septal branch in the ischaemic process and demonstrates that the observed R wave increase is not related to LSFB.

Both the ECG with and without ischaemia show right bundle branch block (RBBB). The usual configurations of this conduction disorder comprise qR, rSR′, RsR′, rsR′ patterns in V1. As the first septal branch supplies the RB, an occlusion including this vessel leads to loss of the initial r wave and then typically shows a QR pattern with a wide Q wave [4]. Given the absence of the QR configuration in V1 and the presence of RBBB already in the ECG without ischaemia, this conduction disorder is not ischaemia related. This is a further argument against involvement of the first septal branch and therefore the septal fascicle.

Regarding the ischaemic ECG, the authors report disappearance of septal Q waves in leads V5 and V6. However, their Fig. 1c in the ‘Answer’ section shows a wide (albeit flat) q wave in V5 (and the same in leads V2 and V3 in Fig. 1b, left panel) [2].

Excluding the possibility of LSFB as the cause of the observed R wave increase in the precordial leads and given the occurrence of wide q waves and notching of the QRS complexes in these similar leads, this phenomenon is more likely explained by local ischaemia-induced conduction delay leading to unopposed anterior wall activation, as previously described, both experimentally and clinically [5].
